# National Association of Dental Faculties

**Published:** 1888-11

**Authors:** 


### THE NATIONAL ASSOCIATION OF DENTAL FACULTIES.

The fifth annual session of this association was held in the Galt House parlors, Louisville, Ky., Aug. 27th, 28th and 29, 1888. The following colleges were represented:

Baltimore College of Dental Surgery, M. W. Foster and B. Holley Smith; Boston Dental College, J. A. Follett; Chicago College of Dental Surgery, A. W. Harlan, T. W. Brophy and J. N. Crouse; Harvard University, Dental Department, Thos. Fillebrown; Kansas City Dental College, J. D. Patterson; Missouri Dental College, W. H. Eames; New York College of Dentistry, Frank Abbott; Ohio College of Dental Surgery, H. A. Smith and G. Mollineaux; Pennsylvania College of Dental Surgery, C. N. Pierce; Philadelphia Dental College, S. H. Guilford; University of Iowa, Dental Department, A. O. Hunt, L. C. Ingersoll and I. P. Wilson; University of Michigan, Dental Department, J. Taft and N. S. Hoff; University of Pennsylvania, Dental Department, Jas. Truman and E. T. Darby; Vanderbilt University, Dental Department, W. H. Morgan and Henry W. Morgan; Northwestern College of Dental Surgery, F. H. B. McDowell, E. J. Perry and N. J. Roberts; Louisville College of Dentistry, A. Wilkes Smith and J. Lewis Howe; Indiana Dental College, Junius E. Cravens; University Dental College of Northwestern University, John S. Marshall; Dental Department of Southern Medical College, L. D. Carpenter; Dental Department of University of Tennessee, J. Y. Crawford; School of Dentistry of Meharry Medical Department of Central Tennessee College, G. W. Hubbard; Dental Department, National University of Washington, D. C., S. J. Cockerill.

The last named college was admitted to membership during the meeting, and completes the list of twenty-three colleges, only one of which was not represented, viz.: The University of California, Dental Department. Two Colleges, the Minnesota Hospital College, Dental Department, and the St. Paul Medical College, Dental Department, had dissolved during the year; and from their two faculties has been formed the Dental Department of the University of Minnesota at Minneapolis. This and the Dental Department of Howard University were proposed for membership; but, under the rule adopted in 1887, their application had to lay over one year. The meeting was called to order by the president, Dr. A. O. Hunt, of the Dental Department of Iowa University, with Dr. J. E. Cravens, of the Indiana Dental College, acting as Secretary. The question arising with regard to the presence of reporters in a secret session, this difficulty was overcome by a motion to allow the Secretary to appoint as many assistants as were required. Dr. Cravens then proceeded to appoint as clerical assistants the representatives of the several journals present, who were not members of the association. While the committee on order of business was preparing to report, the question of credentials was brought up and fully discussed. From the remarks made it was evident that only a small proportion of those present were empowered to act for their institutions. This difficulty will be remedied another year by complying with a resolution offered by Dr. Brophy, later in the session, to the effect that " hereafter a delegate representing a college of the association shall be a member of the teaching faculty of such college, and shall present properly executed credentials specifying his authority to represent his college, before he shall be allowed to vote on questions before the association." As it was, if any decided action had been taken at the meeting, but few of the colleges would have felt themselves bound by the action of their representative in case this action did not suit the majority of the faculty. We consider the resolution given above as one of the most essential passed at the meeting. If the colleges live up to it and pass on the resolution offered by Dr. Eames, " that it is the sense of the meeting that the course of instruction in all colleges in this association should be three years of not less than five months each ; and that the delegates shall submit the proposition to their respective faculties, and report their action to this association at its next annual meeting, in order that a decision on this question may be had," then we may look forward to some definite action being taken on the question at the next meeting of the association.

### A Body Without Authority.

As matters stood, it was evident to all present that the association was little more than an educational institution, where questions relating to the welfare of dental education could be discussed and resolved about; and that aside from the moral influence little else could be accomplished. Our advice in the August PRACTITIONER of leniency towards those schools, that had not lived up to the strict interpretation of the rules and regulations of previous years, was taken, and a probable rupture in one or two directions thus averted. In fact, the association was in no condition to inflict discipline upon any institution, since it is neither an incorporated body, nor even a convention that required credentials in order to gain membership. The extention of the length of college courses was the principle topic of discussion. There seemed to be a general impression that it had to come, and that right early ; but just how or when was the open question. The meeting had a decidedly beneficial effect upon the members present, and it seemed a pity that the entire teaching faculty of the country could not be present and listen or take part in the discussions, because no accurate idea of the character of the meeting can be formed from verbal or printed reports. There was a favorable feeling towards the movement, and if any common plan can be agreed upon, we believe that it will be adopted. An apparently insurmountable difficulty in the way of extending the length of the college term, so as to embrace the spring term, arises from the fact that a large number of colleges are departments of medical colleges, in which the term of instruction is only five months. A lengthened term would, of course, make it necessary for the medical men, who hold chairs in the dental faculties, to give a longer course of instruction to the dental than to their medical classes, unless the extended time should be given entirely to clinical and laboratory work. These institutions would much rather extend the time to three years of five months each, than to two of seven or nine months each. On the whole we were favorably impressed with the evident turn the question is taking. What is wanted is to extend the time for laboratory work, as well as for study; for it is evident that manual training is one of the essential features of a dentist's education, and that this can be obtained only by actual time spent in the laboratory and the operating room. A course of study embracing three years of five months each will give more time to the study of the subject than will two years of seven or even nine months each. By the former plan the student will have three winters and two summers ; while, by the latter, he will have only two winters and one summer.

A Graded Course.

If a graded course of study could be adopted and manual training could be fully introduced, it would be a long step in advance; students could be taken one year younger, just after leaving the grammar school, before habits of study have been broken off, and could spend the first year in the laboratories and in the study of manipulation, as taught in schools for manual training. This instruction could of course be modified from the present form so as to suit the particular needs of dentists. An instructive lesson was brought out by the roll-call as to what instructions the several representatives had received regarding the matter, and as to what the colleges of those who were not instructed would probably agree to. Nine out of eighteen answering either had already, or were willing to adopt, a three years' course of from five to nine months each. Seven were in favor of extending the present course of two years to six, seven or nine months, and in the case of the New York college to twelve months; no one, however, seemed to understand just what Dr. Abbott meant. Five colleges were absent or failed to report, viz.: Boston Dental College, Philadelphia Dental College, Vanderbuilt University, Dental Department, University Dental College of Northwestern University, and University of California, Dental Department. The last two named had no representatives present when the vote was taken. It will be seen from the above table that only two of those that expressed themselves were not in favor of an extension of the length of time. The following is the list as voted by each institution:

| | *Time.* | *Length of Session.* |
|---|---|---|
| Harvard University, Dental Department.... | 3 years | 9 months. |
| Kansas City Dental College.............. | 2 " | 6 " |
| Missouri Dental College.................. | 3 " | 7 or 9 " |
| Ohio College of Dental Surgery..... ...... | 2 " | 6 " |
| Pennsylvania College of Dental Surgery.... | 2 " | 7 " |
| University of Michigan, Dental Department.. | 3 " | 9 " |
| University of Penna., Dental Department.... | 2 " | 7 " |
| Northwestern College of Dental Surgery.... | 3 " | 9 " |
| Louisville College of Dentistry............ | 3 " | 5 " |
| University of Tennessee, Dental Department.. | 2 " | 5 " |
| National University, Dental Department.... | 2 " | 6 " |

The representatives of the following colleges stated that while they have not been instructed, they felt justified, from what they knew of the feeling of their respective faculties, in announcing their positions on the subject as follows:

| | *Time.* | *Length of Session.* |
|---|---|---|
| Baltimore College of Dental Surgery. .....2 | years | 7 months. |
| Indiana Dental College.......... .... ..3 | " | 6 " |
| School of Dentistry, Meharry Medical College, 3 | " | 5 " |
| New York College of Dentistry....... ....2 | " | 12 " |
| Dental Departm't of Southern Medical College, 2 | " | 5 " |
| Chicago College of Dental Surgery ........3 | " | 6 or 9 " |
| State University of Iowa, Dental Department, 3 | " | 5 " |

During the forepart of the session considerable reserve was shown by the members about committing themselves; but when once the ice was broken a perfect storm of resolutions was presented looking toward lengthening the course of instruction. The members vied with one another to see who could get his resolution in first; but as there was a standing rule necessitating all resolutions that embodied radical changes to lie over one year, a large number that obtained seconds were laid over. We give a few of these in order to show the spirit of the meeting. The chairman of the Executive Committee, Dr. Taft, offered the following resolution:

*Whereas*, It is the sense of this Association that less than two years of study and instruction is insufficient properly to prepare any one for the practice of dentistry as demanded by the progress of the times; therefore be it

*Resolved*, That at least two years of *bona fide* study and attendance upon two full regular courses of instruction, in separate years, be required before graduation.

*Mr. McDowell* then offered the following as a substitute for the resolution of the executive committee:

*Resolved*, That after the close of the scholastic year, 1889 and 1890, attendance upon three regular winter courses of instruction of not less than six months each, held in separate years, be required of students by colleges in this association, before examination can be had for graduation.

*Dr. Howe* offered these resolutions:

*Resolved*, To substitute for resolution adopted in N. Y., 1884, the following: "Attendance upon three full regular courses, of not less than five months each, in separate years, shall be required before examination for graduating; and no student shall be graduated until at least twenty-eight months after his first matriculation."

*Resolved*, To amend resolution adopted N. Y., 1884, by inserting in the place of "one year's privilege in a dental office," the words "one course of lectures in a dental college."

*Resolved*, That recommendation adopted N. Y., 1884, "That three years' study of dentistry, including attendance upon two regular courses of lectures, be required previous to examination for graduation," be made mandatory.

*Dr. James Truman* offered the following:

*Whereas*, The present confused condition of dental education in this country, having largely grown out of immature ideas in regard to proper modes of training, as well as modes of expediency; and

*Whereas*, It is vital to the best interests of our profession that we should constantly keep in view that time, repetition of ideas and application combined alone ensure success in any calling, and

*Whereas*, The profession, through its organized association, has demanded of this association that it raise the standard of training. Be it therefore

*Resolved*, That in compliance with this forcibly expressed desire, and in conformity with our own convictions, this association decrees that the sessions of the colleges under its jurisdiction shall, at the close of the coming sessions of 1888 and 1889, be lengthened to at least seven months; and be it further

*Resolved*, That in the event of the adoption of the foregoing resolution, the spring course of lectures be abolished.

It will thus be seen that the spirit of progress is abroad, and that next year we may hope to see some marked step taken in the direction of higher education.

### An Earnest Meeting.

We were impressed with the evident sincerity shown by the members in their desire to formulate some plan upon which all could unite for the common good. All seemed to appreciate the need of some co-operative action. Inability to force any movement was only too evident, since the association is held together by bonds too frail for any of its members or even for a majority of them to adopt aggressive measures. It is an educational institution, and must use only such means to inculcate its measures as will foster and not drive out its membership. It is an institution where the representatives of the several faculties can get together, compare notes, and suggest methods of advancement in matters of dental education. While widely differing views were, in some instances, expressed; yet the utmost good feeling prevailed between the members throughout the meeting. Still, we do not want to be deceived by this apparant harmony and outward expression of intentions on the part of the association as a body, for the adoption of rules looking towards the advancement of the standard of dental education. There was no excuse for anything else but harmony. No positive steps were taken, nor indeed could have been taken under two years, that would directly affect the present regulations which the colleges are following. Even if all the colleges take action on Dr. Eames' resolution the present winter, and send representatives to the next meeting, authorized to vote for a three years' course of five months each, which is highly improbable. It is our opinion that there will be a sufficient number who will refuse, claiming that they had sent out their announcements for the year 1889 and 1890, and that it is too late for the new regimé to take effect until 1890 and 1891.

This is putting the matter in its most favorable aspect; for if only a few colleges, say one or two of the strongest colleges numerically, shall refuse to enter into the arrangement and be bound by its action, then all farther legislation will be blocked, for there is no power to force them into it. This is shown in the case of the University of Maryland, which to-day stands as a serious menance towards the action of the association. The other colleges will refuse to act for fear of the competition of these institutions.

### The Way Out.

It is evident that the only way the National Association of Dental Faculties has to enforce its action is through the National Association of Dental Examiners. They have the law on their side, and have almost arbitrary power as to what institutions, the degrees of which, they shall recognize.

If the National Association of Dental Examiners had at the Louisville meeting passed a resolution to the effect that, after July 1, 1889, the diploma of no college would be recognized that did not require attendence up to, at least, fifteen months of instruction, not including supplementary spring courses, unless such courses were made obligatory. This would have had a most salutary effect, and a large per cent. of the colleges would, irrespective of any action on the part of the National Association of Dental Faculties, adopt either a two years' course of seven and one-half months each, or a three years' course of five months each. During the coming session a test case of the power of the National Association of Dental Examiners to force an institution into line will no doubt be made of the University of Maryland, Dental Department. We wait with considerable interest the result. In the mean time the good work of educating the several faculties as to the need of longer time is going on, and cannot help but have a beneficial influence. Several of the colleges have already taken this step, and we predict that before any decided action has been taken by the National Association of Dental Faculties, a respectable number, if not a majority, will have adopted an extension of time for the course of study. This whole movement may, however, be precipitated by the action of the National Board of Dental Examiners at its next session. We hope that the several State Boards will, during the coming winter, act on the matter, and send their delegates to the next meeting prepared to adopt some regulations looking towards the solution of the problem.

